# Modulating Yogurt Fermentation Through Pulsed Electric Fields and Influence of Milk Fat Content

**DOI:** 10.3390/foods14111927

**Published:** 2025-05-29

**Authors:** Graciela A. Miranda-Mejía, Anaberta Cardador-Martínez, Viridiana Tejada-Ortigoza, Mariana Morales-de la Peña, Olga Martín-Belloso

**Affiliations:** 1 Tecnologico de Monterrey, Escuela de Ingeniería y Ciencias, Ave. Eugenio Garza Sada 2501, Monterrey 64849, NL, Mexico; a01206309@tec.mx (G.A.M.-M.); viri.tejada@tec.mx (V.T.-O.); 2 Tecnologico de Monterrey, Escuela de Ingeniería y Ciencias, Epigmenio González 500, Querétaro 76130, Mexico; mcardador@tec.mx; 3 Department of Food Technology, Engineering and Science, Universitat de Lleida, Rovira Roure 191, 25198 Lleida, Spain; 4 Agrotecnio Center, Rovira Roure 191, 25198 Lleida, Spain

**Keywords:** pulsed electric fields, yogurt, fermentation, lactic acid bacteria

## Abstract

Yogurt is a highly consumed dairy product valued for its nutritional and probiotic properties. Its production involves the use of lactic acid bacteria, which drive biochemical transformations during fermentation. Optimizing fermentation time without compromising yogurt quality is essential for improving processing efficiency. Pulsed electric fields (PEFs) constitute a promising technology that stimulates microbial activity. In this study, a yogurt starter inoculum suspended in milk (IM) with different fat content (0.5–2.8%) was treated with low-intensity PEFs (1 kV/cm, 800–1600 µs) to enhance fermentation kinetics. pH, soluble solids, lactose, lactic acid, and riboflavin concentrations were monitored during 6 h, comparing PEF-treated IM (PEF-IM) and untreated IM (C-IM). PEF-treatments applied to IM reduced the fermentation time of inoculated milk by 4.3–20.4 min compared to C-IM. The lowest fermentation time (5.1 ± 0.16 h) was observed in milk added with PEF-IM (2.8% fat) treated at 1 kV/cm for 1600 µs. Milk inoculated with PEF-IM exhibited enhanced lactose consumption (1.6–3.1%) and higher lactic acid production (7.2%) than milk with C-IM. Riboflavin concentration (0.9–7%) decreased between 2 and 4 h, but it stabilized at the end of fermentation. Obtained results suggest that PEFs promote reversible electroporation in microbial cells, facilitating nutrient uptake and acidification, making it a promising assisted-fermentation approach to improve yogurt production.

## 1. Introduction

Among dairy products obtained through fermentation, yogurt is highly demanded for its nutritional, functional, and sensory characteristics [1,2]. Yogurt is produced by inoculating milk with lactic acid bacteria (LAB), specifically *Streptococcus thermophilus* (*S. thermophilus*) and *Lactobacillus delbrueckii* subsp. *bulgaricus* (*L. bulgaricus*), which exhibit a symbiotic relationship. *S. thermophilus* initiates the fermentation by producing formic acid and CO_2_, which stimulate *L. bulgaricus* growth. In turn, *L. bulgaricus* hydrolyzes milk proteins and releases peptides and amino acids, promoting the growth of *S. thermophilus* and contributing also to yogurt flavor and texture [3,4,5]. These microorganisms metabolize lactose into lactic acid, which causes a drop in pH and generates protein coagulation. Yogurt is considered ready once the inoculated milk achieves a pH range between 4.5 and 4.7 (cut-off pH), ensuring its desired consistency, flavor, and microbial stability [3,4,5]. In addition to acidification, LAB also influences the nutritional profile of yogurt. Some strains can synthesize B-group vitamins such as riboflavin (vitamin B_2_), while others may uptake available riboflavin from the milk during fermentation, depending on their genetic characteristics and physiological state, thereby modulating the final vitamin content in yogurt [6,7].

Milk composition, particularly its fat content, also plays a key role in the fermentation performance. Higher fat concentration is associated with slower acidification rates due to the buffering capacity of fat globules and the reduced availability of aqueous-phase nutrients. Nevertheless, milk fat improves texture and sensory attributes and may also contribute to LAB stability under processing conditions [8].

Fermentation is one of the most energy-demanding and time-consuming stages in yogurt production, usually requiring between 4 and 6 h under standard industrial conditions. Therefore, finding alternative approaches able to improve fermentation efficiency without compromising product quality, represents a great challenge for the food industry [9,10]. In this context, novel technologies have gained attention as promising processes to assist fermentation, showing numerous advantages. Among them, pulsed electric fields (PEFs) are recognized as a promising non-thermal process characterized by their short processing time, from micro to milliseconds, and high potential for enhancing microbial performance when applied at low intensity. Reversible electroporation induced by low-intensity PEFs (0.1–3 kV/cm) has been reported to transiently increase microbial membrane permeability. This facilitates greater nutrient uptake and accelerates metabolic activity, promoting faster fermentation without compromising cell viability [11,12,13]. Regarding milk fermentation, electroporation may enhance the metabolic activity of *S. thermophilus* and *L. bulgaricus* by improving their uptake of lactose and micronutrients, which are essential for acid production and symbiotic growth [14].

Within the last decade, PEF processing (0.2–3.67 kV/cm) of yogurt starter cultures has been studied to improve fermentation kinetics without compromising product quality. PEF treatment of *S. thermophilus* and *L. bulgaricus* has been shown to enhance acidification kinetics in reconstituted skimmed milk, reducing the pH lag phase by 12 min [15]. Similarly, improvements in the fermentation rate, texture, and reduction of syneresis have also been observed when mixed LAB cultures were suspended in peptone water and exposed to PEFs [16]. Recently, applying PEFs to LAB inoculated into whole milk significantly shortened total fermentation time by 31.2 min without compromising the physicochemical or sensory quality of the obtained yogurt after 14 days of refrigerated storage [11].

On the other hand, it has been reported that PEF effects on microorganisms are highly influenced by the composition of the medium ant its physicochemical properties, such as conductivity [17,18,19]. Despite this, based on the current available literature, no studies have addressed how the treatment of the inoculum by PEFs and medium characteristics affect LAB performance during milk fermentation to obtain yogurt.

Hence, the aim of this study was to assess the effect of applying low-intensity PEFs at different treatment times on inoculum suspended in milk (IM) with 0.5 and 2.8% fat content, by monitoring the fermentation time and analyzing changes in the lactose, lactic acid, and riboflavin content of milk supplemented with the IM throughout the fermentation process.

## 2. Materials and Methods

### 2.1. Yogurt Manufacturing and Determination of Fermentation Endpoint

The procedure to obtain natural yogurt was conducted according to the method previously established in Miranda-Mejía et al. [11]. Initially, the inoculum was made under sterile conditions in a laminar flow hood, using 800 mL of commercial UHT (ultra-high temperature) treated milk with 0.5 or 2.8% fat content (Alpura, Querétaro, Mexico) and 200 g of commercial thermophilic culture composed of *S. thermophilus* and *L. bulgaricus* (Novonesis, Bagsvaerd, Capital region, Denmark; culture YF-L904, batch No: 3751231, MEX), reaching an initial microbial load of 2.4 × 10^9^ CFU/mL. The mixture of starter culture and milk with different fat content (IM-0.5 and IM-2.8) was blended at 150–170 rpm for 20 min to ensure homogeneity. Subsequently, 330 μL of IM-0.5 or IM-2.8 was transferred into 330 mL of milk with 0.5 or 2.8% fat content, respectively, gently mixed, and incubated at 42 °C for 6 h [20,21]. The pH was monitored every hour until reaching the cut-off pH (4.7), which marked the end of the fermentation and yogurt formation.

An acidification curve was plotted using pH values vs. time obtained during 6 h of yogurt fermentation. Then, the fermentation time was calculated by solving *x* with Wolfram Alpha (Wolfram Research, Champaign, IL, USA) from Equation (1):

(1) $$y=x^{2}-x+a$$

where *y* corresponds to the cut-off pH (4.7), *a* is a constant parameter, and *x* denotes the fermentation endpoint.

### 2.2. Pulsed Electric Field Treatment

PEF treatment was carried out using an EPULSUS^®^-LPM1A-10 system (Energy Pulse Systems, Lisbon, Portugal) with a maximum output of 10 kV, 200 A, and 3 kW. The equipment featured a batch parallel treatment chamber comprising two stainless steel electrodes separated by a 10 cm gap and insulated with a nylon dielectric material, operating in monopolar mode. The IM-0.5 or IM-2.8 were subjected to PEF treatment before adding them to the milk, when microorganisms were in the lag phase. IM (80 mL) with electrical conductivity values of 22.9 mS/cm (0.5% fat content) and 18.5 mS/cm (2.8% fat content) were located inside the treatment chamber and PEF processed during different treatment times (*t*). *t* was determined by multiplying the pulse number by the pulse width (µs). The temperature of IM was taken before and right after PEF treatment using a digital thermometer (PECULA TP101, Shenzhen, China). Under all tested conditions, the temperature remained below 24 ± 0.5 °C. To independently assess the effect of the PEF, untreated IM (C-IM) was used as a control and subjected to the same handling procedures without PEF application, ensuring all other fermentation conditions were identical. PEF-treated IM (PEF-IM) and C-IM were added to the milk and incubated under the same conditions to monitor fermentation kinetics.

### 2.3. Experimental Design

A 2^3^ factorial experiment was set up to investigate the influence of milk fat content (0.5 and 2.8%) and PEF processing at an electric field strength (*E*) of 1 kV/cm, pulsed with (τ) of 8 µs, and a frequency (*f*) of 10 Hz during different *t* (800, 1200, and 1600 µs) on microbial performance through fermentation. The selection of these conditions was based on prior research and preliminary experiments [11]. A total of six PEF treatments were applied to IM as indicated in Table 1, having the milk with C-IM as reference.

The evaluation of lactose, lactic acid, and riboflavin concentration changes during fermentation was performed on the milk added with PEF-IM, achieving the lowest fermentation time (PEF-IM_OPT_), comparing results with milk containing C-IM. This evaluation allowed a comparative analysis of fermentation biochemical changes between milks with PEF-IM at optimum conditions and C-IM.

### 2.4. pH, Total Soluble Solids, and Conductivity Measurements

Measurements of total soluble solids (°Brix) and pH were performed on 20 mL of IM with a refractometer (HANNA HI 96813, HANNA Instruments, Woonsocket, RI, USA) and a potentiometer (OAKTON pH 510, OAKTON Instruments, Vernon Hills, IL, USA), respectively. Conductivity was measured (80 mL) using a conductivity meter (OAKTON 6+, OAKTON Instruments, Vernon Hills, IL, USA).

### 2.5. Microbial Count

Bacterial count was performed according to Kang et al. (2019) with some modifications [22]. The culture medium was prepared by dissolving 70 g of MRS (de Man–Rogosa–Sharpe) (DIFCO, Becton, Dickinson, and Company, Sparks, MD, USA) agar in 1 L of distilled water, sterilized (121 °C/15 min at 15 psi), and poured into Petri dishes. The plates were left to solidify at room temperature for 40 min, covered with parafilm, and placed in storage upside down under refrigerated conditions (4 ± 1 °C) until use. IM were stirred at 160 rpm for 1 h to ensure a homogeneous mixture. Six serial dilutions were prepared, inoculated in petri dishes, and incubated 24 h at 42 ± 0.5 °C. Bacterial colonies were enumerated with a digital colony counter, and the number of colony-forming units per milliliter (CFU/mL) was calculated with Equation (2).

(2) $$\frac{CFU}{mL}=\frac{Averages\;colony\;count}{Dilution\;factor\times volume\;plated}$$

### 2.6. Lactose and Organic Acids Extraction and Quantification

Lactose and organic acids extractions were performed following the procedure of Leclercq-Perlat (1999) with some modifications [23]. Milks with PEF-IM_OPT_ and C-IM (750 µL for lactose and 1000 µL for organic acids) were blended with 1 mL of deionized water and placed in a water bath (50 °C) for 1 h. After incubation, the mixtures were vortexed at 160,000 rpm for 2 min and immediately cooled using an ice bath to approximately reach 25 °C. For lactose extraction, 1250 µL each of Carrez I and Carrez II reagents, along with 250 µL of sodium hydroxide, were incorporated into the mixture. In the case of organic acid extraction, 1 mL of a 240 g/L trichloroacetic acid solution (SIGMA-Aldrich, St. Louis, MO, USA) and an additional 1 mL of distilled water were added. Both extracts were subsequently vortexed again for homogenization and incubated for 1 h at 25 °C. The resulting solutions were filtered through 0.45 µm PTFE membranes (Agilent Technologies, Santa Clara, CA, USA) and transferred into 2 mL Eppendorf tubes. Lactose (L_e_) and Organic acid (OA_e_) extracts were preserved at 4 °C and analyzed within 15 days to avoid degradation. To minimize analyte degradation and ensure consistency across all extracts, the procedures were carried out using freshly prepared reagents. Extractions were performed under chilled conditions, and the contact time with Carrez and TCA solutions was standardized to reduce variability caused by exposure duration.

For HPLC analysis, L_e_ and OA_e_ were transferred to HPLC vials and identified following the procedure described by Picque et al. (1993) with some modifications [24]. Vials were injected in an Agilent 1200 series system (Agilent Technologies, Santa Clara, CA, USA) equipped with a refractive index detector (RID) with an optical temperature of 30 °C for lactose (Lac), and a diode array detector (DAD) for (LA), propionic (PA), and butyric (BA) acids. A Zorbax Eclipse XBD-C18 column (4.6 × 150 mm, 5 µm, Agilent, Santa Clara, CA, USA) maintained at 50 °C was used to separate the compounds with an isocratic flow rate of 1 mL/min of the mobile phase (5 mM sulfuric acid) for 15 min. A constant injection volume of 10 µL was used for all analytes and standards. Identification of Lactose (Lac) was based on the retention time and RID spectral comparison with 10 g/L of standard (SIGMA-Aldrich, St. Louis, MO, USA). Lactic acid (LA), propionic acid (PA), and butyric acid (BA) were identified by comparing their UV-Vis spectra and retention times with certified reference standards: LA (40 g/L), PA (20 g/L), and BA (20 g/L), all from SIGMA-Aldrich (St. Louis, MO, USA). The quantification of Lac was based on the peak integration of RID signals, whereas organic acids were quantified by integrating the peak area at 210 nm. Calibration curves were prepared for each compound obtaining R^2^ values of 0.9997 for Lac, 0.9952 for LA, 0.9962 for PA, and 0.9762 for BA. Results were presented as the percentage of lactose and organic acids in 100 mL of milk added with IM.

### 2.7. Riboflavin Extraction and Quantification

Riboflavin (vitamin B_2_) extraction and quantification were performed according to the procedure of Albalá-Hurtado et al. (1997) and Gliszczyńska-Świglo and Koziolowa (2000) with some modifications [25,26]. Milks with PEF-IMOPT and C-IM (1050 µL) were transferred to 50 mL centrifuge tubes and blended with 0.1 g of trichloroacetic acid (TCA, SIGMA-Aldrich, St. Louis, MO, USA). The mixture was agitated on a magnetic stirring plate (CIMAREC) for 10 min and then centrifuged at 1250× *g* for 10 min (MULTIFUGE X1R, Thermo Scientific, Waltham, MA, USA). After collecting the supernatant, the remaining pellet was re-extracted adding 300 µL of 4% TCA, stirred (10 min), and centrifuged under the same conditions. The solid phase was discarded, and both supernatants were combined in a 2 mL amber Eppendorf tube, adjusting the volume with 4% TCA. The extract was passed through a 0.45 µm PTFE filter (Agilent Technologies, GER, Santa Clara, CA, USA) and kept in amber vials (4 °C) until it was analyzed. Throughout the extraction and filtration steps, ambient light exposure was minimized by covering the centrifuge tubes with aluminum foil.

Riboflavin analysis was conducted in an Agilent 1200 series HPLC system (Agilent Technologies, Santa Clara, CA, USA) equipped with a fluorescence detector (FLD) set at 35 °C. Separation of riboflavin was performed by injecting 20 µL of riboflavin extract into the system, using a Tracer Spherisorb ODS2-C18 column (4.6 × 250 mm, 5 µm, Agilent, Santa Clara, CA, USA) set at room temperature (25 °C), with an isocratic elution of 0.7 mL/min of the mobile phase during a 20 min runtime. The mobile phase was prepared by first dissolving 1.8 g of octane sulfonic acid (OSA, SIGMA-Aldrich, St. Louis, MO, USA) in 800 mL of double-distilled water. Then, 24 mL of glacial acetic acid (MEYER, Mexico City, Mexico) and 5 mL of triethylamine (SIGMA-Aldrich, St. Louis, MO, USA) were added to the solution, which was thoroughly mixed. The pH was adjusted to 3.6 ± 0.1 using either glacial acetic acid to lower the pH or triethylamine to raise it, depending on the initial value. Once the target pH was achieved, 150 mL of LC-grade methanol (SIGMA-Aldrich, St. Louis, MO, USA) was added. The solution was mixed again and volumetrically filled to one liter with double-distilled water. Finally, it was filtered using a 0.45 µm membrane.

Riboflavin identification was conducted by comparing the retention time and fluorescence signal with those of the standard solution (100 mg/L riboflavin, SIGMA-Aldrich, GER, St. Louis, MO, USA), which was prepared in aqueous acetic acid (2.4%, *v*/*v*). Quantification was performed by integrating the areas of the obtained peaks at 450 nm (excitation wavelength) and 525 nm (emission wavelength). Data were compared to a calibration curve, which showed a correlation coefficient of R^2^ = 0.9811. The riboflavin content was reported in percentage per 100 mL of milk containing IM.

### 2.8. Statistical Analysis

PEF experiments were carried out in duplicate, and each treatment was analyzed in triplicate. Statistical differences between group means were evaluated using Tukey’s post-hoc test and an independent Student’s *t*-test. A significance threshold of *p* < 0.05 was applied to determine significant differences between treatments. All statistical evaluations were conducted using Minitab software (Minitab, LLC, State College, PA, USA, Version 19.2020.10).

## 3. Results and Discussion

### 3.1. Effect of PEF Treatment on Fermentation Process

#### 3.1.1. Fermentation Time

A progressive pH decline from 6.63 ± 0.01 (0 h) to 4.48 ± 0.01 was observed during the fermentation process of milk with C-IM. The cut-off pH (4.7) was reached at 5.9 ± 0.07 h in C-IM0.5 and at 5.4 ± 0.18 h for C-IM2.8. These fermentation time are within the typical yogurt fermentation time range (5.0s–6.5 h) reported by other authors [11,27]. The observed changes in pH are associated with the consumption of fermentable carbohydrates, mainly lactose, by the LAB during fermentation [24].

**Figure 1:**
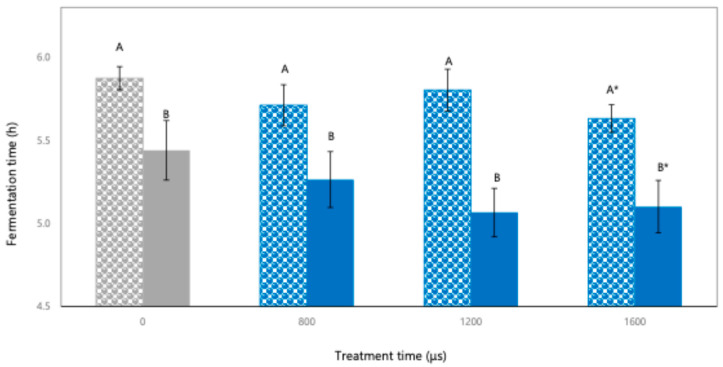
Fermentation time (h) of milk with PEF treated inoculum (PEF-IM) at different treatment times (800, 1200, and 1600 µs) having milk with untreated inoculum (C-IM) as control (0 µs, gray bars). Dotted-patterned bars correspond to milk with 0.5% fat content, and solid bars to milk with 2.8% fat content. A, B indicate significant differences (*p* < 0.05) between matrices at the same treatment time. * indicates significant differences (*p* < 0.05) within the same matrix at different treatment times. Treatment codes correspond to those listed in Table 1.

As shown in Figure 1, milks with PEF-IM (0.5 and 2.8% fat content) at different treatment times exhibited a reduction in fermentation time compared to milk with C-IM. A longer PEF treatment time consistently resulted in a shorter fermentation time, regardless of milk fat content. According to Miranda-Mejía et al. (2024) and Chanos et al. (2020), this effect might be attributed to the reversible electroporation in LAB induced by the PEF processing at low electric field intensity (1 kV/cm) for long treatment times, enhancing cell membrane permeability [11,15]. This mechanism may facilitate the lactose uptake of microorganisms from the media, converting it to lactic acid and accelerating LAB metabolism [28]. Although post-treatment microbial viability was not directly quantified, the accelerated acidification and reduction in fermentation time observed in milks with PEF-IM suggest that the LAB remained functionally active and that PEFs may have enhanced their metabolic performance. These results are in agreement with previous studies reporting that low-intensity PEFs (1–3 kV/cm) can stimulate LAB through reversible electroporation, increasing nutrient transport and promoting lactic acid production without compromising cell viability [16,29]. In addition to electroporation, a sublethal cellular stress response might be triggered during PEF processing, stimulating the metabolic activity of LAB, shortening the lag phase, and enhancing acidification. Therefore, the combination of membrane permeabilization and controlled stress induction at 1 kV/cm appeared to enhance lactose metabolism and lactic acid production by the LAB during fermentation [15,30].

Interestingly, milk with PEF-IM0.5 resulted in a longer fermentation time compared to milk with PEF-IM2.8, irrespective of the processing time. This trend could be explained by the higher concentration of phospholipids, lipophilic vitamins, and other bioactive lipids in milk with a higher fat content (2.8%), which might protect LAB and contribute to membrane stabilization, improving microbial tolerance to PEF-induced stress [31,32]. In contrast, milk with a lower fat content (0.5%) lacks these protective compounds, which may limit bacterial adaptation, particularly under longer treatments. Despite these compositional differences, both inoculated milk matrices were added with the same concentration of starter culture, achieving an initial bacterial population of ~2.4 × 10^9^ CFU/mL. This indicates that milk fat content did not influence the initial microbial viability before PEF application. This is consistent with previous studies reporting that variations in milk fat content do not significantly alter the initial survival counts of starter cultures when inoculated at fixed proportions, especially in UHT milk systems [5,33,34]. Therefore, the differences observed during the fermentation of milk with PEF-IM could be attributed to the matrix-dependent response to electroporation and conductivity properties, rather than the initial microbial load. Notably, electrical conductivity differed significantly between matrices. IM with 2.8% fat content exhibited a moderate conductivity (~18.5 mS/cm), whereas IM with 0.5% fat content showed higher values (~22.9 mS/cm). While moderate conductivity enhances electric field propagation and electroporation efficiency, excessively high conductivity may lead to energy dispersion and localized overheating, potentially compromising microbial performance [28].

Among all evaluated conditions, IM with 2.8% fat content, PEF-treated with pulses of 8 µs at 1 kV/cm during 1600 µs and 10 Hz (PEF-IM2.8-1600) was the most effective treatment, resulting in the greatest fermentation time reduction (20.4 min). These findings confirm that the interaction between PEF parameters, milk fat content, and conductivity plays a critical role in enhancing fermentation kinetics through synergistic effects on LAB metabolic performance.

#### 3.1.2. Changes in Soluble Solids, Lactose, and Organic Acids During Fermentation as Affected by PEF_OPT_

##### Soluble Solids

**Figure 2:**
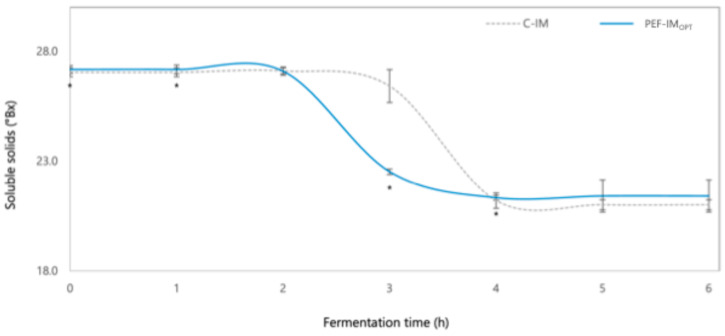
Soluble solids (°Brix) values during fermentation of milk with inoculum treated with PEF at optimal conditions (PEF-IM_OPT_) and untreated inoculum (C-IM). * corresponds to a statistical difference (*p* < 0.05) between milk with C-IM and PEF-IM_OPT_.

Changes in soluble solids (Brix) during the fermentation of milk with PEF-IM_OPT_ and its corresponding C-IM are presented in Figure 2. During the first 2 h, soluble solids remained constant at approximately 27 °Brix in both milks with PEF-IM_OPT_ and C-IM, which is the initial soluble solid content of whole milk (2.8% fat content). Between hours 2 and 4, a clear decrease in soluble solids was observed, especially in milk with PEF-IM_OPT_. In this period, the soluble solids dropped from 27.0 to 23.0 in milk with PEF-IM_OPT_, while in the milk with C-IM, they decreased slightly, from 27.1 to 26.4. These differences suggest that PEF enhanced the activity of LAB, possibly by improving membrane permeability and promoting faster sugar uptake [11,13]. From hour 4 onward, the values stabilized around 21 °Brix in both milks with PEF-IM_OPT_ and C-IM. This result indicates that PEFs influenced the rate at which sugars were consumed, particularly during the early stages of fermentation, without altering the final soluble solids level [16,29].

##### Lactose and Organic Acids

**Figure 3:**
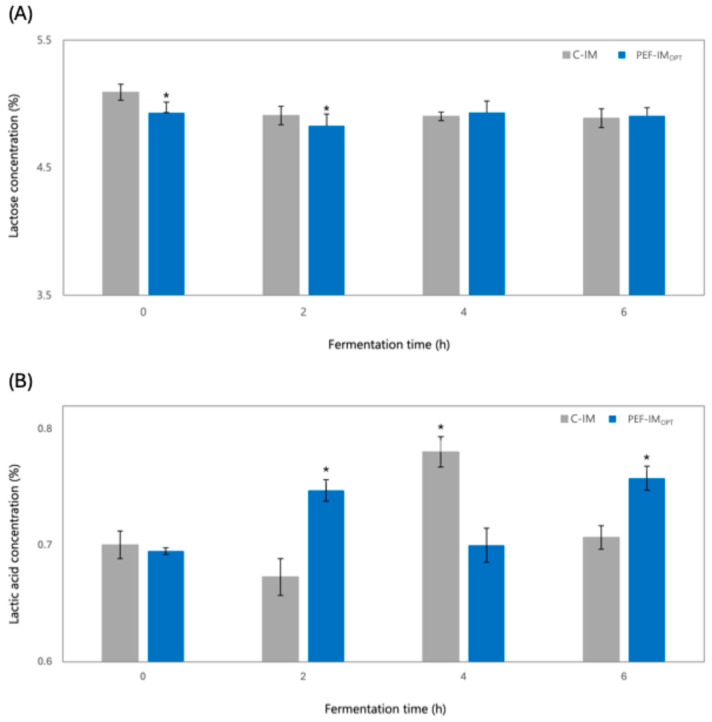
Changes in lactose (**A**) and lactic acid (**B**) concentration of milk with PEF treated IM at optimal conditions (PEF-IM_OPT_) and untreated inoculum (C-IM) during fermentation. * corresponds to a statistical difference (*p* < 0.05) between the CIM and PEF-IM_OPT_.

The lactose concentration showed a gradual decline during fermentation in milk supplemented with PEF-IM_OPT_, with the most pronounced reduction occurring during the first 2 h (Figure 3A). At both 2 and 4 h, lactose content in milk with PEF-IM_OPT_ was significantly lower by approximately 1.6 to 3.1% than in the milk with C-IM, indicating a faster substrate consumption of LAB during the early phase of fermentation. This suggests that PEF treatment to IM before the fermentation stage enhanced the metabolic activity of LAB, likely by increasing membrane permeability and stimulating nutrient transport systems, as previously reported [13]. From hour 4 onwards, lactose concentrations stabilized in both milks, with PEF-IM_OPT_ and C-IM, indicating that most of the lactose transformation reactions occurred during the early fermentation phase. These results support the hypothesis that low-intensity PEFs accelerate the rate but not the extent of carbohydrate utilization, promoting a faster transition through the exponential growth phase of LAB. The accelerated lactose depletion observed in milk with PEF-IM could be linked to PEF effects on enzymatic activity. Although the precise molecular mechanisms remain under investigation, it has been proposed that PEFs may enhance the function of lactose permeases (LacS) and β-galactosidase (LacZ), facilitating more efficient substrate uptake and hydrolysis during early fermentation [35]. Consistently, previous studies have shown that PEF treatment enhanced enzymatic activity and acid production in *L. bulgaricus* without compromising cell viability [29].

As expected, LA concentrations increased progressively throughout fermentation in milk supplemented with PEF-IM_OPT_ and C-IM (Figure 3B). At 2 and 6 h of fermentation, milk with PEF-IM_OPT_ exhibited significantly higher LA content than milk supplemented with C-IM, which was 11% higher at 2 h and 7.2% higher at 6 h (*p* < 0.05), indicating that low-intensity PEF treatment enhanced early metabolic activity of LAB and sustained acid production until the end of fermentation. These results align with the reduction observed in total soluble solids and pH, suggesting an accelerated onset of carbohydrate metabolism [11]. Interestingly, at the 4th hour of fermentation, LA concentration in milk with C-IM was 10.3% higher than in the milk supplemented with PEF-IM_OPT_ (*p* < 0.05). This transient drop in LA accumulation in the milk with PEF-IM may reflect a brief physiological adjustment of the LAB following PEF exposure. Previous studies have shown that reversible electroporation induced by low-intensity PEF can temporarily alter membrane permeability, potentially disrupting intracellular processes such as acid synthesis [29,36]. However, the increased LA content in milk with PEF-IM_OPT_ by 6 h suggests that the LAB successfully adapted to this transient stress and re-established their acidification capacity. A comparable recovery pattern has been observed, indicating that the metabolic activity of LAB can normalize after an initial lag period post-PEF [16]. Likewise, previous studies have reported increased acidification rates and enzymatic activity in *L. bulgaricus* after PEF exposure, along with a faster pH drop and greater LA accumulation in yogurt fermented with PEF-treated cultures [11,29].

Moreover, no traces of propionic or butyric acids were detected in either milk with PEF-IM_OPT_ or milk with C-IM throughout fermentation. This indicates that the starter cultures exhibited selective metabolic activity favoring lactic acid production, with no signs of contamination by undesirable bacteria. The lack of these organic acids is considered favorable, as they are often associated with off-flavors such as rancid, bitter, or pungent notes in dairy products [37,38,39]. These findings suggest that PEFs enhance lactic acid production without altering the profile of secondary organic acids, as supported by previous reports [11].

##### Riboflavin

**Figure 4:**
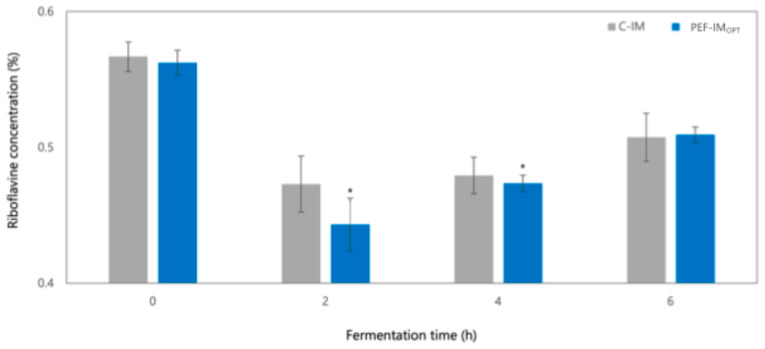
Changes in riboflavin concentration in milk with PEF-treated inoculum at optimal conditions (PEF-IM_OPT_) and untreated inoculum (C-IM) during fermentation. * corresponds to a statistical difference (*p* < 0.05) between the C-IM and PEF-IM_OPT_.

Riboflavin (vitamin B_2_) was evaluated during fermentation in milks with PEF-IM_OPT_ and C-IM to monitor how riboflavin concentration changed as a potential response of LAB to PEFs. Figure 4 presents the concentration of riboflavin in both milks with PEF-IM_OPT_ and C-IM at 0, 2, 4, and 6 h of fermentation. A noticeable decrease in riboflavin content was observed between 2 and 4 h of fermentation in both milks, being 17% lower in milk with PEF-IM_OPT_ compared to milk with C-IM at hour 2. After this drop, riboflavin concentrations stabilized by hour 6, with no significant differences between the two inoculated milks. These findings suggest that PEFs did not affect the final riboflavin content in the obtained yogurt but may have transiently influenced its concentration during the early fermentation phase [40,41].

The temporary decrease in riboflavin content on milk supplemented with PEF-IM_OPT_ may be explained by the reversible electroporation effect induced by low-intensity PEFs, which increases membrane permeability in LAB, potentially enhancing the uptake of extracellular compounds such as riboflavin [40,41]. Additionally, some studies have shown that the metabolic change in riboflavin depends on the strain-specific behavior of LAB. During fermentation, some strains can synthesize riboflavin de novo, while others can uptake it directly from the media depending on their genetic makeup and physiological state [7,42]. Given the observed reduction in riboflavin concentration in milk with PEF-IM_OPT_ at the 2nd hour of fermentation, it is possible that the LAB strains from the starter culture used it as a nutrient for fermentation reactions, especially under PEF-induced stress. By the end of fermentation, the stabilization of riboflavin content in both milks with PEF-treated or untreated IM confirmed that PEF application did not negatively compromise the vitamin’s final concentration in the obtained yogurt. Rather than altering the nutritional quality of the product, PEFs may transiently affect vitamin availability during early microbial adaptation, highlighting the complexity of microbial responses to non-thermal technologies such as PEFs [16,40,41].

Although this study provides insights into the effects of PEFs on vitamin dynamics during fermentation, comprehensive evaluations of the combined impact of PEFs and milk composition remain limited and, to the best of the authors’ knowledge, there is not related information available in the literature. Hence, PEF effects on microbial vitamin uptake mechanisms and its interaction with matrix components in dairy systems warrant further investigation.

## 4. Conclusions

This study demonstrates that PEF treatment applied for a longer time to IM with 2.8% fat content significantly reduced the fermentation time of inoculated milk compared to a conventional process. Adding PEF-IM treated at 1 kV/cm for 1600 µs to the milk showed the greatest effect, reaching the cut-off pH (4.7) to obtain yogurt up to 20.4 min earlier than milk with untreated IM. The reduction in fermentation time on milk with PEF-IM was associated with enhanced LAB metabolic activity, likely due to induced membrane permeability leading to a faster lactose depletion and lactic acid accumulation. Furthermore, moderate conductivity values (~18.5 mS/cm) of treated media could promote a uniform electric field propagation during PEF processing, improving the electroporation effect. Although riboflavin levels showed a temporary decline during early fermentation in milk with PEF-IM, final concentrations remained stable, confirming that PEFs preserved the nutritional quality of the obtained yogurt. Overall, low-intensity PEFs demonstrated potential as a viable non-thermal strategy to improve yogurt process efficiency by accelerating milk fermentation without adversely affecting the physicochemical characteristics and vitamin content of the obtained yogurt. Further studies are needed to deepen our understanding of the microbial response to PEFs and to evaluate its scalability for industrial applications.

## Figures and Tables

**Table 1:** PEF treatments (1 kV/cm, pulse width 8 µs at 10 Hz) applied to inoculated milk (IM) with different fat content (0.5 and 2.8%).

Treatment	Fat (%)	*t* (µs)
C-IM0.5	0.5	0
PEF-IM0.5-800		800
PEF-IM0.5-1200		1200
PEF-IM0.5-1600		1600
C-IM2.8	2.8	0
PEF-IM2.8-800		800
PEF-IM2.8-1200		1200
PEF-IM2.8-1600		1600

Inoculum size: 200 g of starter culture in 800 mL of milk.

## Data Availability

The original contributions presented in the study are included in the article. Further inquiries can be directed to the corresponding authors.

## Abbreviations

The following abbreviations are used in this manuscript:

PEF	pulsed electric fields
IM	inoculum suspended in milk
PEF-IM	PEF-treated IM
C-IM	untreated-IM, as control
LAB	lactic acid bacteria
IM-0.5	inoculum suspended in milk with 0.5% fat content
IM-2.8	inoculum suspended in milk with 2.8% fat content
PEF-IMOPT	PEF-IM at optimum conditions
MRS	de Man–Rogosa–Sharpe
*t*	treatment times
CFU/mL	colony forming units per milliliter
Le	lactose extracts
OAe	organic acid extracts
RID	refractive index detector
Lac	lactose
DAD	diode array detector
LA	lactic acid
PA	propionic acid
BA	butyric acid
HPLC	high-performance liquid chromatography
TCA	trichloroacetic acid
FLD	fluorescence detector
OSA	octane sulfonic acid
C-IM0.5	untreated-IM, as control with 0.5% fat content
C-IM2.8	untreated-IM, as control with 2.8% fat content
